# Quercetin potentially suppresses HBV activity through targeting the RIG-I/MAVS pathway

**DOI:** 10.1016/j.isci.2026.115704

**Published:** 2026-04-12

**Authors:** Yan Yang, Chen Luo, Yan Liu, Lian Xia, Yunhao Yang, Junyuan Deng, Daike Zou, Chenglin Tang, Xiaohe Xiao, Feilin Ge

**Affiliations:** 1 Department of Liver Diseases, Chongqing Traditional Chinese Medicine Hospital, Chongqing 400021, China; 2 College of Acupuncture and Tuina, Chongqing University of Chinese Medicine, Chongqing 402760, China; 3 The Fifth Medical Center, Chinese PLA General Hospital, Beijing 100039, China; 4 Department of Chinese Medicine, State Key Laboratory of Antiviral Drugs, The First Affiliated Hospital of Zhengzhou University, Zhengzhou 450052, China

**Keywords:** Cell biology, Molecular biology

## Abstract

Quercetin exhibits potent anti-hepatitis B virus (HBV) activity through a host-directed mechanism involving innate immune activation. In this study, quercetin was evaluated in a panel of *in vitro* and *in vivo* HBV infection models, where it effectively suppressed HBV replication and reduced viral antigen expression. Mechanistically, quercetin activated the RIG-I/MAVS signaling pathway, thereby enhancing downstream antiviral signaling and promoting interferon (IFN)-β production. Molecular interaction analysis and RIG-I knockdown experiments further demonstrated that quercetin directly binds to RIG-I and exerts its antiviral effects in a RIG-I-dependent manner. These findings establish the quercetin-induced activation of the RIG-I/MAVS axis as a key mechanism underlying its anti-HBV activity and highlight quercetin as a promising host-targeting candidate for HBV treatment.

## Highlights

• Quercetin suppresses HBV and drug-resistant HBV *in vitro* and *in vivo*
• Quercetin promotes antiviral signaling and IFN-β production
• Quercetin directly binds RIG-I to enhance host innate immunity
• Quercetin may serve as a lead compound for host-directed anti-HBV therapy

## Graphical abstract

**Figure:**
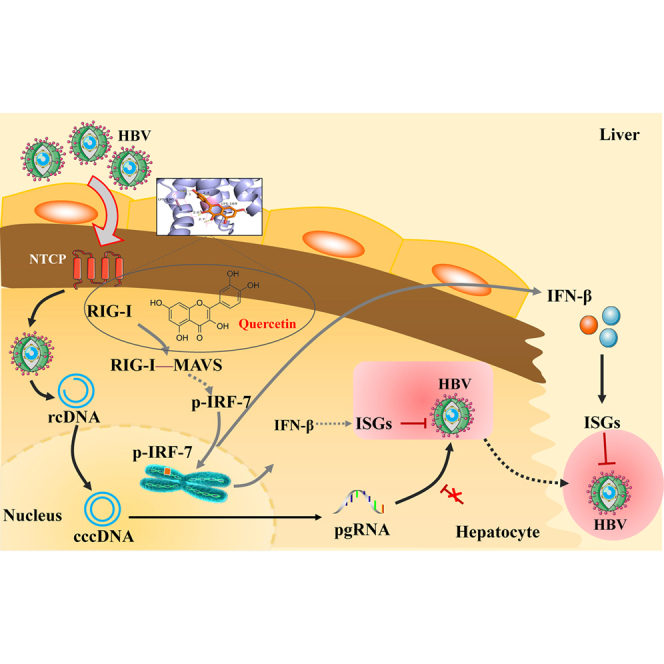

## Introduction

Hepatitis B virus (HBV) infection leads to chronic hepatitis B (CHB) and significantly increases the risk of severe liver diseases, including cirrhosis, liver failure, and hepatocellular carcinoma (HCC).^1^ This poses a major threat to global public health. Although the widespread administration of the hepatitis B vaccine has reduced the incidence of acute and chronic HBV infections, the disease remains a persistent global public health challenge. In 2019, the World Health Organization (WHO) reported a global prevalence of 3.8% for HBsAg in the general population, with approximately 296 million individuals suffering from chronic infection and 1.5 million new HBV infections.^2^ Clinical first-line anti-HBV drugs consist of two main classes: interferon (IFN), an immunomodulator, and nucleotide analogs (NAs) that inhibit the activity of HBV polymerase/reverse transcriptase.^3^ Due to a low response rate and patient tolerance issues, Peg-IFN-α has limited clinical utility. Consequently, NAs, such as tenofovir disoproxil fumarate (TDF), entecavir (ETV), and the recently approved tenofovir alafenamide fumarate (TAF) and tenofovir methoxypropyl fumarate (TMF), represent the most widely used second-generation oral reverse transcriptase inhibitors in clinical practice for anti-HBV treatment. Nevertheless, NAs only inhibit viral replication without achieving HBsAg loss, necessitating long-term or lifelong treatment for patients.^4^ Achieving a functional cure for HBV infection, characterized by negativity in both HBV DNA and HBV antigens, is the most desirable therapeutic outcome for patients with CHB. Hence, the quest for drugs capable of compensating for the limitations of NAs holds clinical significance in promoting functional HBV cure.

The interaction between HBV and the host immune system plays a critical role in determining clinical outcomes and disease progression during chronic infection. The innate immune system not only recognizes and inhibits viral replication but also crucially regulates the adaptive immune response. Therefore, harnessing the innate immune response represents a key strategy for achieving a functional cure for CHB.^1^ In a prospective cohort study, IFN agents modulating natural immunity achieved functional cure in 45% of patients with CHB.^5^ HBV infects cells and produces single-stranded RNA, double-stranded rcDNA/cccDNA, and double-stranded RNA during replication, and these nucleic acids exposed in the cytoplasm or entered into the intracellular body are recognized by various pattern recognition receptors, ultimately triggering IFN production for antiviral effects.^6^ The TLR pathway and RIG-I pathway are classical antiviral signaling pathways within the natural immune system, playing crucial roles in antiviral defense. In summary, there is a strong rationale for exploring drug development aimed at promoting functional cure of CHB through the activation of the innate immune response.

Previous studies from our group have demonstrated that quercetin, a small molecule derived from traditional Chinese medicine, effectively inhibits HBV replication, particularly with respect to viral antigen suppression.^3^ The underlying mechanism was suggested to involve the regulation of innate immune responses. In addition, accumulating evidence indicates that quercetin exhibits broad biological activities, including anti-inflammatory, antioxidant, antitumor, immunoprotective, and antiviral effects.^7^^,^^8^^,^^9^^,^^10^^,^^11^ Nevertheless, although several studies have reported the anti-HBV activity of quercetin, most of these investigations primarily focused on its direct antiviral effects, whereas studies addressing immune-mediated anti-HBV mechanisms remain relatively limited.^12^^,^^13^^,^^14^^,^^15^^,^^16^ Moreover, the mechanistic understanding of quercetin-mediated HBV inhibition is still incompletely explored.

**Figure 1:**
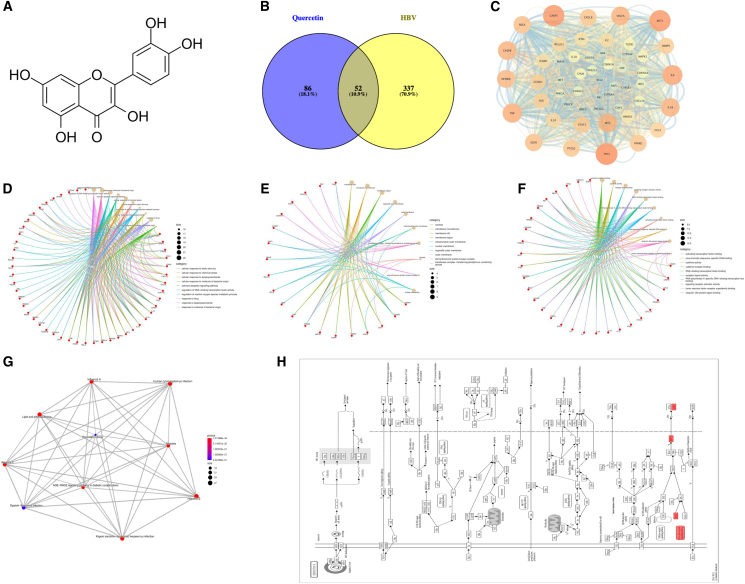
Network pharmacology analysis of quercetin in the treatment of HBV infection (A) The chemical structure of quercetin. (B) Venn diagram shows the intersection between quercetin-related targets and HBV-related genes. (C) PPI network of intersecting targets. (D–F) GO enrichment analysis results of the intersecting targets: (D) biological process (BP), (E) cellular component (CC), and (F) molecular function (MF). (G) KEGG pathway enrichment analysis. (H) KEGG KO-based pathway map of HBV signaling.

Based on these findings, this study comprehensively evaluates the anti-HBV effects of quercetin using both *in vivo* and *in vitro* HBV models. A combination of network pharmacology, molecular docking, molecular dynamics simulation, and multiple molecular biology techniques was employed to elucidate the role of RIG-I pathway activation in quercetin’s anti-HBV activity (Figure 1). The ultimate goal is to provide valuable lead compounds for the development of novel therapeutics that aim to achieve a functional cure for CHB. According to the TCMSP database, quercetin is a key active constituent in at least 188 traditional Chinese medicines, underscoring its broad potential for pharmaceutical development. Thus, this work also provides a foundation for advancing quercetin-containing herbal formulations.

## Results

### Network pharmacology analysis identifies potential targets of quercetin against HBV

The chemical structure of quercetin is shown in Figure 1A. A total of 138 putative quercetin-related targets were retrieved from the TCMSP database, while 389 HBV-related targets were obtained from the GeneCards database. Venn analysis revealed 52 overlapping targets (Figure 1B), suggesting their potential involvement in the anti-HBV activity of quercetin.The common targets were imported into the STRING database to construct a PPI network, which revealed key nodes such as MAVS and IRF7 (Figure 1C). GO enrichment analysis was significantly enriched for biological processes includingresponse to virus, immune system regulation, and cytokine-mediated signaling pathways (Figures 1D–1F; Tables S1–S3). KEGG pathway enrichment indicated that the shared targets were mainly enriched in viral infection-related and immune signaling pathways.Notably, the hepatitis B pathway was among the top enriched terms(Figure 1G; Table S4). Further KO-based KEGG pathway analysis revealed that these targets occupy key positions in the HBV-associated signaling network, especially in components related to innate immunity and antiviral responses (Figure 1H). Importantly, the significant enrichment of the RIG-I-like receptor signaling pathway strongly suggests that quercetin may exert its anti-HBV effects primarily by modulating this host antiviral innate immune pathway.

### Cytotoxicity and antiviral effects of quercetin on HepG2.2.15 and HepG2.A64 cells

**Figure 2:**
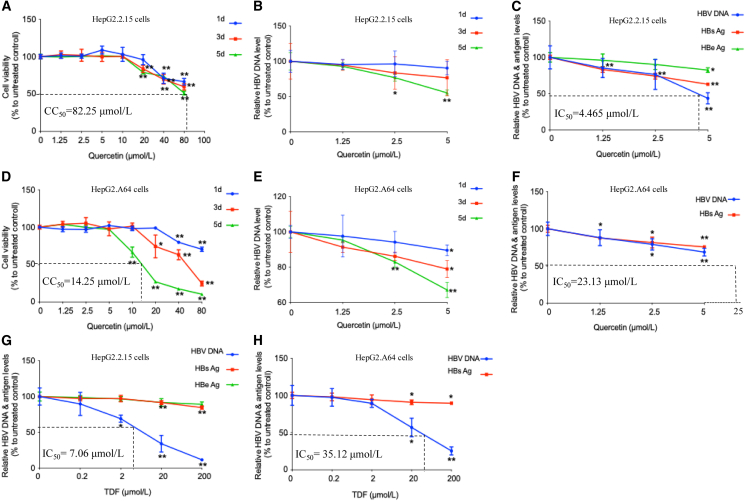
Evaluation of quercetin safety, efficacy duration, and antiviral effects *in vitro* (A and D) Cell viability assays were performed to determine the safe concentration of quercetin in HepG2.2.15 (A) and HepG2.A64 (D) cells. (B and E) The optimal effective duration of quercetin treatment was assessed in HepG2.2.15 (B) and HepG2.A64 (E) cells. (C and G) Antiviral efficacy of quercetin and TDF was evaluated in HepG2.2.15 cells by measuring HBV DNA, HBsAg, and HBeAg levels in the supernatant. (F and H) Antiviral efficacy of quercetin and TDF in HepG2.A64 cells was assessed by measuring HBV DNA and HBsAg levels in the supernatant. The data were presented as mean ± standard deviation (SD). ∗*p* < 0.05 and ∗∗*p* < 0.01 compared to the untreated control group.

The CCK-8 assay for quercetin revealed no cytotoxicity at 10 μmol/L, and the CC_50_ was 82.25 μmol/L in HepG2.2.15 and 14.25 μmol/L in HepG2.A64 (Figures 2A and 2D). Based on this safe concentration, the anti-HBV effect of quercetin was evaluated over different treatment durations (days 1, 3, and 5). Quercetin exhibited the most potent antiviral activity after 5 days of treatment in both cell lines(Figures 2B and 2E). In wild-type HBV-replicating cells (HepG2.2.15), quercetin (5 μmol/L) exhibited inhibition rates of 56.30%, 36.92%, and 17.44% on HBV DNA, HBsAg, and HBeAg, respectively (Figure 2C). Simultaneously, TDF (200 μmol/L) exhibited inhibition rates of 88.43%, 15.48%, and 10.87% on HBV DNA, HBsAg, and HBeAg, respectively (Figure 2G). Furthermore, in ETV-resistant HBV-replicating cells (HepG2.A64), quercetin (5 μmol/L) demonstrated inhibition rates of 31.35% and 24.47% on HBV DNA and HBsAg, respectively (Figure 2F). Concurrently, TDF (200 μmol/L) displayed inhibition rates of 74.59% and 10.48% on HBV DNA and HBsAg, respectively (Figure 2H).

### Antiviral effects of quercetin on HBV-infected mice

**Figure 3:**
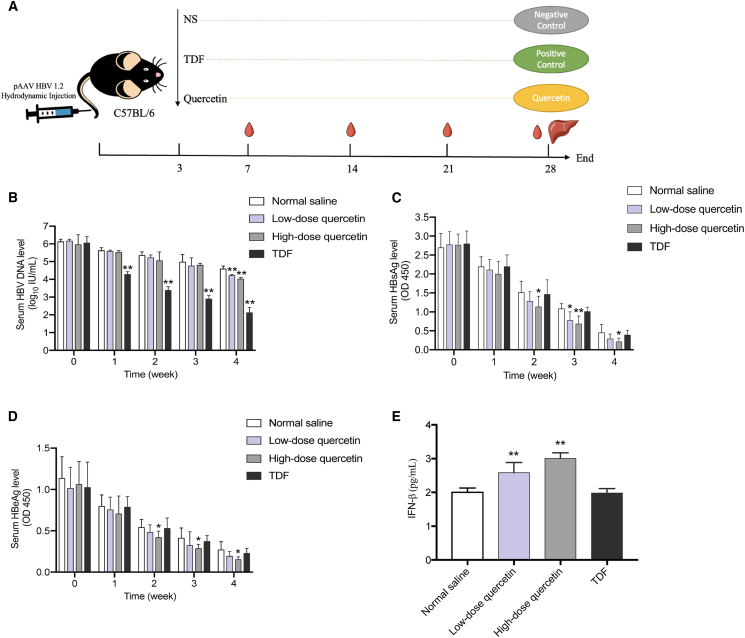
Quercetin reduces HBV replication and enhances IFN-β production *in vivo* (A) Schematic of the *in vivo* experimental design. (B–D) Serum levels of HBV DNA (B), HBsAg (C), and HBeAg (D) were measured weekly. (E) Serum IFN-β levels were determined at the 4th week. The data were presented as mean ± SD. ∗*p* < 0.05 and ∗∗*p* < 0.01 compared to the Normal saline.

A hydrodynamic injection-based HBV replication mouse model was established in C57BL/6 mice (Figure 3A). HBV DNA levels in the low-dose (37.5 mg kg^−1^ d^−1^) and high-dose (75 mg kg^−1^ d^−1^) quercetin groups, as well as the TDF (63 mg kg^−1^ d^−1^) group, exhibited significant reductions of 0.37, 0.57, and 2.47 log_10_ IU/mL, respectively, in comparison to the NS group. The results indicated that the inhibitory effect of quercetin on *in vivo* HBV DNA synthesis increased with dose, although it was slightly lower than that of TDF (Figure 3B). Serum HBsAg and HBeAg levels were also markedly reduced after quercetin treatment, particularly in the high-dose group (Figures 3C and 3D). Furthermore, serum IFN-β levels increased by 1.29-fold, 1.50-fold, and 0.99-fold in low-dose quercetin, high-dose quercetin, and TDF-treated mice, respectively (Figure 3E), suggesting that quercetin may activate antiviral innate immune responses.

**Figure 4:**
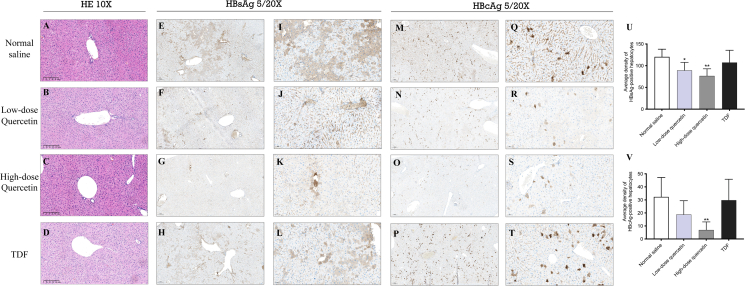
The Effect of quercetin on hepatic virology in HBV-Infected mice (A–D) Representative images of HE staining showing liver tissue morphology across groups. (E–L) Immunohistochemical staining of HBsAg. (M–T) Immunohistochemical staining of HBcAg. (U and V) Quantification of positive hepatocytes for HBsAg and HBcAg, respectively. Scale bars, 200 μm. The data were presented as mean ± SD. ∗*p* < 0.05 and ∗∗*p* < 0.01 compared to the normal saline.

Although HE staining revealed no significant histopathological differences among the groups(Figures 4A–4D), immunohistochemical analysis demonstrated a marked reduction in HBV antigen-positive hepatocytes following quercetin treatment compared to the TDF and NS groups (Figures 4E–4T). Quantitative analysis showed that the average densities of HBsAg-positive hepatocytes in the NS-, TDF-, low-dose quercetin-, and high-dose quercetin-treated groups were 120.43 ± 17.64, 107.40 ± 28.21, 89.35 ± 18.38, and 76.58 ± 16.39, respectively (Figure 4U). Likewise, the average densities of HBcAg-positive hepatocytes were 32.36 ± 14.84, 29.82 ± 16.03, 18.82 ± 10.58, and 6.92 ± 6.17 in the NS-, TDF-, low-dose quercetin-, and high-dose quercetin-treated groups, respectively (Figure 4V). Notably, HBsAg- and HBcAg-positive hepatocytes were predominantly distributed within the hepatic lobular parenchyma, without an apparent preferential localization to either the periportal or centrilobular regions. The primary differences among treatment groups were reflected in the number and density of antigen-positive hepatocytes rather than in their spatial distribution patterns. These results indicate that quercetin treatment, particularly at high dose, effectively reduced HBV antigen expression in liver tissue.

### Regulation of the RIG-I/MAVS signaling pathway by quercetin

**Figure 5:**
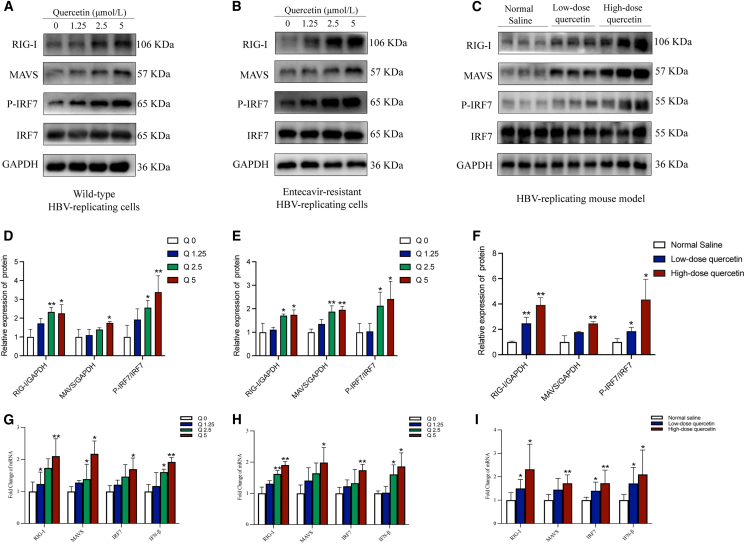
Quercetin exerts its antiviral effects by activating the RIG-I/MAVS signaling pathway (A–C) Western blot analysis of RIG-I, MAVS, and *p*-IRF7 protein levels in HepG2.215 cells (A), HepG2.A64 cells (B), and liver tissues of HBV-infected mice (C), GAPDH was used as the loading control. (D–F) Quantification of protein expression levels shown in A–C, respectively. (G–I) qPCR analysis of mRNA levels of RIG-I, MAVS, IRF7, and IFN-β in HepG2.215 cells (G), HepG2.A64 cells (H), and liver tissues of HBV-infected mice (I). The data were presented as mean ± SD. ∗*p* < 0.05 and ∗∗*p* < 0.01 compared to the untreated control or Normal saline. All *in vitro* experiments were independently repeated three times (*n* = 3), and representative blots are shown.

To further investigate the mechanism by which quercetin exerts antiviral effects against HBV, we examined its regulatory impact on the RIG-I signaling pathway both *in vivo* and *in vitro*. Western blot analysis demonstrated that quercetin treatment significantly increased the protein expression levels of *p*-IRF7, RIG-I, and MAVS. This upregulation exhibited a dose-dependent trend, with higher quercetin concentrations leading to greater induction of pathway proteins (Figures 5A–5F). Consistent with the protein data, RT-qPCR results showed that quercetin markedly elevated the mRNA expression levels of RIG-I, MAVS, IFN-β, and IRF7. Among these, RIG-I and IFN-β showed the most significant increases (Figures 5G–5J). The degree of upregulation was positively correlated with the quercetin dose, suggesting enhanced activation of the antiviral immune response through the RIG-I signaling.

### Molecular docking and dynamics simulation of quercetin with RIG-I

**Figure 6:**
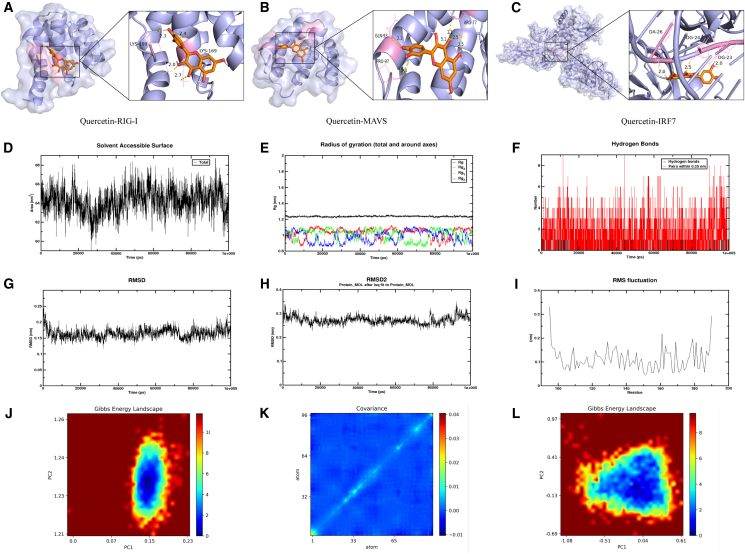
Molecular docking and molecular dynamics analysis of quercetin with RIG-I (A–C) Molecular docking conformations of quercetin with RIG-I, MAVS, and IRF7. (D) Interaction interface area of the quercetin–RIG-I complex, used to evaluate interface stability and its dynamic changes. (E) Radius of gyration (Gyrate), reflecting the compactness and stability of the overall RIG-I structure. (F) Number of hydrogen bonds (HBnum), indicating whether quercetin forms a stable hydrogen-bonding network with RIG-I. (G and H) Root-mean-square deviation (RMSD and RMSD2), assessing the conformational fluctuations of RIG-I or the complex over time; smaller fluctuations indicate higher stability. (I) Root-mean-square fluctuation (RMSF), showing the flexibility of individual RIG-I residues, with emphasis on fluctuations at the binding site residues. (J) Gibbs free energy (Gibbs), evaluating whether the complex formation is thermodynamically favorable. (K) Intermolecular covalent and noncovalent interaction energies (CovaPic), revealing the major energetic contributions to complex stability. (L) Principal component analysis combined with Gibbs free energy (PCA_Gibbs), visualizing the dominant motion patterns of RIG-I and the influence of quercetin binding.

Molecular docking revealed moderate binding affinities between quercetin and key antiviral proteins, with binding energies of −5.8 kcal/mol for RIG-I, −6.6 kcal/mol for MAVS, and −9.4 kcal/mol for IRF7, indicating stable interactions (Figures 6A–6C); notably, quercetin was predicted to interact predominantly with the CARD domain of RIG-I, involving key residues LYS-169 and LYS-190, providing structural insight into its potential role in RIG-I activation. Molecular dynamics simulation showed that the quercetin-RIG-I complex maintained stable conformations throughout the simulation. SASA values fluctuated between 62 and 68 nm^2^, indicating an initial decrease then increase in hydrophobic surface exposure, suggesting tighter folding (Figure 6D). The radius of gyration (Rg) remained stable around 1.2–1.3 nm, showing no significant expansion or collapse (Figure 6E). Hydrogen bonds between quercetin and RIG-I ranged from 2 to 4, reflecting stable interactions (Figure 6F). Quercetin’s RMSD mostly stayed between 0.15 and 0.2 nm, while the complex RMSD fluctuated near 0.3 nm, both indicating structural stability without major conformational changes (Figures 6G–6I). RMSF analysis showed low backbone fluctuations (<0.2 nm), with minor flexible regions, and stable active site (Figure 6J). Gibbs free energy landscapes confirmed conformational stability without abrupt changes (Figures 6K and 6L).

### Quercetin exhibits anti-HBV activity via RIG-I: Validation by SPR, siRNA, and pAAV-Mediated animal experiments

**Figure 7:**
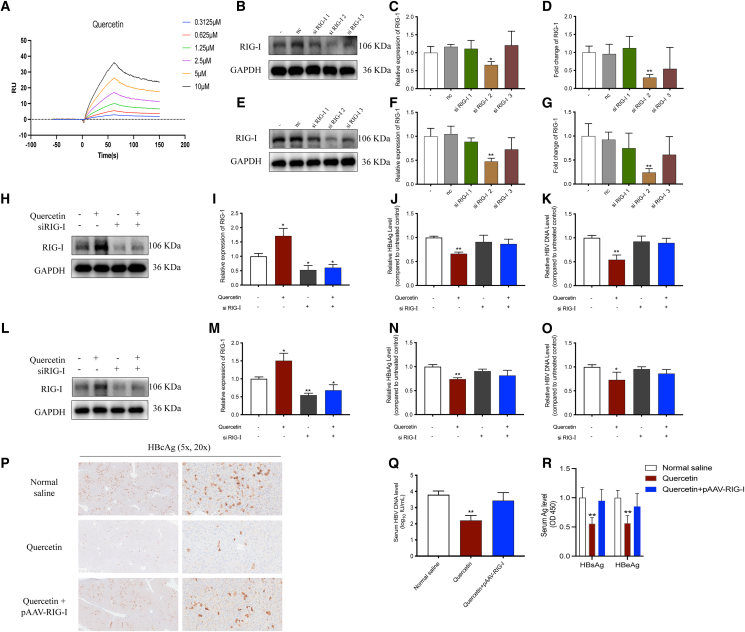
Functional validation of RIG-I as a key mediator of quercetin’s anti-HBV activity (A) SPR assay shows the binding interaction between quercetin and RIG-I. (B–G) Screening and validation of three different siRNAs targeting RIG-I in HepG2.215 and HepG2.A64 cells. Western blot analysis (B, E), corresponding quantification (C, F), and qPCR analysis of RIG-I mRNA expression (D, G). (H, I, L and M) Effects of the selected siRNA (si-RIG-I) combined with quercetin treatment on RIG-I activation in HepG2.215 and HepG2.A64 cells, shown by western blot (H, L) and densitometric analysis (I, M). (J, K, N and O) Effects of si-RIG-I and quercetin co-treatment on HBV replication and antigen expression in HepG2.215 and HepG2.A64 cell supernatants, including quantification of HBsAg (J, N), and HBV DNA (K, O). (P) Immunohistochemical staining of HBcAg. (Q) Serum HBV DNA levels. (R) Serum HBsAg and HBeAg levels. The data were presented as mean ± SD. ∗*p* < 0.05 and ∗∗*p* < 0.01 compared to the untreated control.

**Figure 8:**
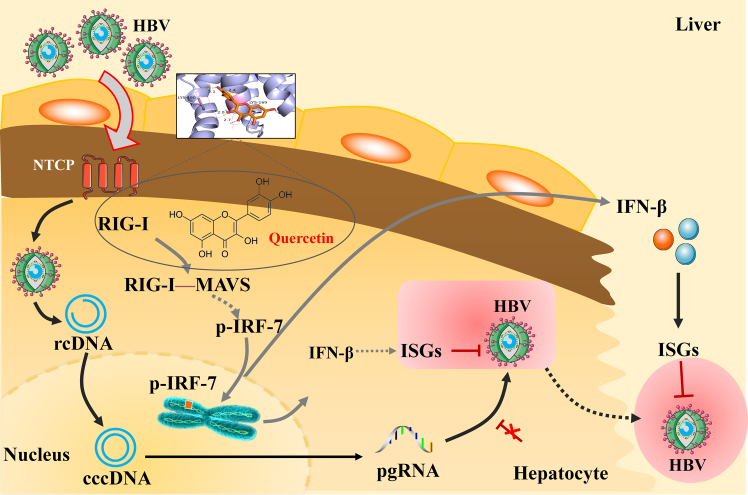
Quercetin exerts its antiviral effect mainly through activation of RIG-I/MAVS

SPR analysis confirmed a direct interaction between quercetin and RIG-I, with a dissociation constant (KD) of 5.72 μM, indicating moderate binding affinity (Figure 7A). To further validate the functional role of RIG-I in quercetin’s antiviral mechanism, three siRNAs targeting RIG-I were screened, and the most effective one (siRIG-I-2) was selected based on knockdown efficiency (Figures 7B–7G). Co-treatment of HBV-infected hepatocyte cell lines with quercetin and siRIG-I significantly reduced RIG-I expression (Figures 8H, 8I, 8L, and 8M) and abolished the antiviral effects of quercetin, as evidenced by increased HBV antigen and HBV DNA levels compared to quercetin treatment alone (Figures 7J, 7K, 7N and 7O). In animal models, RIG-I inhibition mediated by pAAV reversed the suppressive effects of quercetin on HBcAg (Figure 7P), HBV DNA (Figure 7Q), HBsAg and HBeAg (Figure 7R), further confirming the central role of RIG-I in quercetin’s anti-HBV activity. However, given the partial knockdown efficiency, these results indicate that RIG-I is functionally required for quercetin-mediated antiviral effects, rather than serving as the sole determinant.

## Discussion

CHB infection poses a significant threat to global public health. Despite the availability of preventive vaccines, an individual succumbs to HBV-related disease every 30 s worldwide.^17^ Achieving a functional cure is the ideal therapeutic goal for CHB, a goal that is increasingly pursued in clinical practice. The criteria include the sustained disappearance of HBsAg at the end of an effective treatment course, HBV DNA negativity, remission of hepatic inflammation and histopathologic improvement, along with a significant reduction in the incidence of end-stage liver disease.^18^ Despite the high effectiveness of existing first-line therapeutic agents, NAs, in inhibiting HBV DNA, they exhibit limited inhibitory effects on HBV antigens. The constraints of NAs not only impede the functional cure of patients with CHB but also pose a significant obstacle to the WHO goal of “eliminating viral hepatitis as a public health hazard by 2030.”^19^^,^^20^ Numerous studies have demonstrated that the potent clearance of HBV antigens can be achieved through the activation of innate immunity, with IFN playing a central role. This approach represents a key strategy for achieving the functional cure of CHB.^21^ Against this background, the present study aims to explore natural small-molecule compounds that can clear HBV antigens via innate immune activation, with the ultimate goal of identifying candidate or lead compounds capable of advancing CHB functional cure.

Our study demonstrated that quercetin exerted antiviral effects against both wild-type and ETV-resistant HBV. Compared with TDF, quercetin showed superior efficacy in reducing HBV antigen levels, although its inhibitory effect on HBV DNA was relatively weaker. This antiviral activity was further validated in an HBV-replicating mouse model. HBV antigens, such as HBsAg, play a pivotal role in establishing immune tolerance and may contribute to viral persistence by suppressing host immunity through the modulation of the IFN-related pathway.^22^ The limited capacity of NAs to suppress viral antigens has long represented a major bottleneck in achieving a functional cure for CHB. It should be noted that these findings are derived from preclinical models, and their clinical relevance should be interpreted with caution. The superior efficacy of quercetin in inhibiting HBsAg production highlights its potential to act synergistically with NAs in overcoming this therapeutic barrier.

Quercetin may exert its anti-HBV effects through the regulation of the RIG-I/MAVS pathway. Indeed, HBV infection is a dynamic process involving complex interactions between the virus, hepatocytes, and the host immune system. CHB is recognized as both a viral and immunological disease, with immune responses playing a pivotal role in both HBV pathogenesis and therapeutic outcomes. The type I IFN-mediated innate immune response driven by the RIG-I/MAVS signaling pathway is essential for HBV clearance. When the helicase and RD domains of RIG-I recognize and bind viral RNA, the CARD domain is released and subsequently interacts with the adaptor protein MAVS located on the mitochondrial membrane. MAVS then transmits signals to downstream cytoplasmic transcription factors such as IRF7. Upon activation, IRF7 is phosphorylated (P-IRF7) and translocates into the nucleus to initiate IFN transcription.^23^^,^^24^ IFNs are classified into three types according to their receptor specificity: type I (IFN-α, IFN-β), type II (IFN-γ), and type III (IFN-λ). Among these, type I IFNs exhibit broad activity, exerting antiviral effects by restricting viral replication and dissemination in the early phase of infection, thereby constituting a critical component of the host’s innate antiviral defense.^25^ A prospective cohort study further demonstrated that the modulation of innate immunity, particularly via type I IFN therapy, could achieve a functional cure in up to 45% of patients with CHB.^5^ In this study, through integrative bioinformatics analysis and *in vitro/in vivo* experimental validation, we found that quercetin markedly activated the RIG-I/MAVS signaling pathway. These findings suggest that the anti-HBV activity of quercetin is closely associated with the activation of the RIG-I/MAVS axis (Figure 8).

Quercetin may exert its anti-HBV effects by interacting with RIG-I and modulating the RIG-I/MAVS/IRF7 signaling pathway. Type I IFNs represent the cornerstone of antiviral defense against HBV, and RIG-I-like receptors, as critical initiators of type I IFN responses, have emerged as one of the key immunological targets for HBV therapy.^26^ Consequently, the development of RIG-I–based targeted therapies against HBV has remained both a research hotspot and a major challenge. According to the latest pipeline of investigational hepatitis B drugs published on the Hepatitis B Foundation website, seven candidate compounds currently in development aim to activate innate immunity. These agents are primarily TLR agonists and RIG-I agonists, with IFN serving as the ultimate effector, and clinical trial data have demonstrated superior anti-HBV efficacy.^27^ Thus, exploring therapeutic strategies that promote the functional cure of CHB through the activation of innate immune responses is of great importance, supported by robust theoretical rationale and clinical evidence. In this context, our study identified RIG-I as the direct molecular target of quercetin using SPR, molecular dynamics simulations, and siRNA knockdown experiments. Importantly, while RIG-I is identified as a key mediator of quercetin-induced antiviral effects, it should not be considered the exclusive innate immune pathway involved, and the potential contribution of other pattern recognition receptors warrants further investigation in future studies. Furthermore, we explicitly acknowledge that although our findings support quercetin as a candidate compound with a defined molecular target and immunomodulatory potential, whether equivalent or sufficient systemic exposure can be achieved in humans via oral administration remains to be determined through future pharmacokinetic evaluations and formulation optimization. Collectively, these results not only elucidate a key immunological mechanism underlying quercetin’s anti-HBV activity but also highlight its potential as a novel RIG-I–modulating agent contributing to the pursuit of a functional cure for CHB. Moreover, as a key bioactive constituent present in multiple traditional Chinese medicines, quercetin possesses broad translational relevance. The present study provides a valuable mechanistic framework for the further development of quercetin-based herbal therapeutics and host-directed antiviral strategies against HBV.

The RIG-I/MAVS pathway should be regarded as a major, but not exclusive, mechanism underlying the anti-HBV effects of quercetin. Other signaling pathways, including the NF-κB pathway, the Nrf2 pathway, and the JAK1/STAT3/HIF-1α pathway, which have been reported to be modulated by quercetin in different disease contexts,^28^^,^^29^ may also exert synergistic or complementary effects. The potential involvement of these pathways warrants further systematic investigation in future studies.

In conclusion, this study demonstrates that quercetin exerts a promising anti-HBV effect, as validated in both wild-type and drug-resistant HBV cell lines, as well as *in vivo* models. Notably, quercetin surpassed the positive control TDF in suppressing HBsAg production. Mechanistically, its antiviral action appears to involve the activation of the RIG-I pathway, leading to downstream type I IFN. Therefore, quercetin holds potential as a novel RIG-I agonist and may represent a promising therapeutic candidate for CHB treatment.

### Limitations of the study

While our study provides preliminary evidence supporting the anti-HBV potential of quercetin, several limitations should be acknowledged. First, the mechanistic investigation remains insufficiently in-depth, and the precise molecular events underlying the antiviral effects of quercetin require further clarification in future studies. Second, pharmacokinetic characterization of quercetin was not included in the present work, and additional studies are needed to define its absorption, distribution, metabolism, and excretion profiles. Third, omics-based analyses were lacking, which limited our ability to comprehensively delineate the global regulatory networks and biological processes involved. In future work, we will further investigate the molecular mechanisms and pharmacokinetic properties of quercetin, while incorporating multi-omics approaches to provide more robust preclinical evidence for its druggability and therapeutic development.

## Resource availability

### Lead contact

Further information and requests for resources should be directed to and will be fulfilled by the lead contact, Feilin Ge (18810821080@163.com).

### Materials availability

The study did not generate new materials.

### Data and code availability

• All data reported in this paper are included in the article and its supplemental information. This study did not generate any publicly deposited datasets.
• This paper does not report original code.
• Any additional information required to reanalyze the data reported in this paper is available from the lead contact upon request.

## Acknowledgments

This work was financially supported by the 10.13039/501100001809National Natural Science Foundation of China (82404975),the Postdoctoral Science Foundation of China (2024M763015, 2025M783952),The National Funded Postdoctoral Researcher Program of China (no. GZC20232406), the Henan Province Traditional Chinese Medicine Science Research Project (no. 2023ZY3040), and the Henan Province Medical Science and Technology Research Plan Joint Construction Project (LHGJ20230233).

## Author contributions

Y.Y., C.L., and Y.L.: data analysis and drafting the manuscript, revising the manuscript. Y.Y.: data analysis and drafting the manuscript, cell experiments. C.L.: data analysis and drafting the manuscript, animal experiments. Y.L.: data analysis and drafting the manuscript. L.X.: cell experiments. Y.Y., J.D., and D.Z.: network pharmacology analyses, as well as molecular docking and molecular dynamics simulations, and provided critical interpretation of the results. C.T., X.X., and F.G.: manuscript revision, provided constructive comments, and polished the final draft. All data were generated in-house, and no paper mill was used. All authors reviewed, discussed, and approved the final version of the manuscript.

## Declaration of interests

The authors declare that they have no conflict of interests.

## STAR★Methods

### Key resources table

**Table:** 

REAGENT or RESOURCE	SOURCE	IDENTIFIER
**Antibodies**		

m IRF7	Cell Signaling Technology	Cat# 72073, RRID:AB_3073735
h IRF7	Cell Signaling Technology	Cat# 4920, RRID:AB_2127551
m pIRF7	Cell Signaling Technology	Cat# 24129, RRID:AB_2798872
h pIRF7	Cell Signaling Technology	Cat# 5184, RRID:AB_10621425
MAVs	Proteintech	Cat# 66911-1-Ig, RRID:AB_2882238
RIG-I	Proteintech	Cat# 20566-1-AP, RRID:AB_10700006
GAPDH	GeneTex	Cat# GTX100118, RRID:AB_1080976

**Experimental models: Cell lines**		

HepG2.2.15	Institute of Infectious Diseases, Chinese PLA General Hospital	RRID: CVCL_L855
HepG2.A64	Institute of Infectious Diseases, Chinese PLA General Hospital	RRID: CVCL_B0UH

**Experimental models: Organisms/strains**		

C57BL/6 mice	Chongqing Medical University	RRID:MGI:2159769

**Oligonucleotides**		

Primer: Human IFN-β Forward: ATGACCAACAAGTGTCTCCTCC Reverse: GGAATCCAAGCAAGTTGTAGCTC	This paper	N/A
Primer: Human IRF7 Forward: TTGGCTCCTGAGAGGGCA Reverse: TTGGTTGGGACTGGATCTGC	This paper	N/A
Primer: Human RIG-I Forward: CTGGACCCTACCTACATCCTG Reverse: GGCATCCAAAAAGCCACGG	This paper	N/A
Primer: Human MAVS Forward: CAGGCCGAGCCTATCATCTG Reverse: GGGCTTTGAGCTAGTTGGCA	This paper	N/A
Primer: Human si RIG-I Forward: CCACUUAAACCCAGAGACAAUTT Reverse: AUUGUCUCUGGGUUUAAGUGGTT	This paper	N/A
Primer: Human GAPDH Forward: AATGGGCAGCCGTTAGGAAA Reverse: GCCCAATACGACCAAATCAGAG	This paper	N/A
Primer: Mouse IFN-β Forward: CAGCTCCAAGAAAGGACGAAC Reverse: GGCAGTGTAACTCTTCTGCAT	This paper	N/A
Primer: Mouse IRF7 Forward: GAGACTGGCTATTGGGGGAG Reverse: GACCGAAATGCTTCCAGGG	This paper	N/A
Primer: Mouse RIG-I Forward: AAGAGCCAGAGTGTCAGAATCT Reverse: AGCTCCAGTTGGTAATTTCTTGG	This paper	N/A
Primer: Mouse MAVS Forward: CTGCCTCACAGCTAGTGACC Reverse: CCGGCGCTGGAGATTATTG	This paper	N/A
Primer: Mouse GAPDH Forward: AGGTCGGTGTGAACGGATTTG Reverse: GGGGTCGTTGATGGCAACA	This paper	N/A

**Software and algorithms**		

ImageJ	NIH	RRID:SCR_003070
Prism (Graphpad)	GraphPad Prism 9	https://www.graphpad.com/
Adobe Illustrator	Adobe Illustrator (2023)	https://www.adobe.com/creativecloud.html

### Experimental model and study participant details

#### Mice

Male C57BL/6 mice (8 weeks old) were procured from Chongqing Medical University (Chongqing, China). All mice were housed in a specific pathogen-free (SPF) facility under controlled conditions (temperature: 22 ± 2 °C, humidity: 55 ± 10%, 12-h light/dark cycle) with *ad libitum* access to food and water. All animal procedures were approved by the Ethics Committee of Chongqing Medical University (Approval No.: IACUC-CQMU-2024-0911) and conducted in accordance with the institutional guidelines for the care and use of laboratory animals.

#### Cell culture and measurement of cell viability

Two HBV-replicating cell lines, HepG2.2.15 and HepG2.A64, were used in the study.HepG2.2.15 represents a wild-type HBV cell model, while HepG2.A64 is a LAM+ETV drug-resistant mutant HBV stable replicating cell model. Compared to HepG2.2.15 cells, HepG2.A64 cells produced comparable levels of HBV DNA, but higher levels of HBsAg and lower levels of HBeAg. Both cell lines were cultured in DMEM with 2% penicillin/streptomycin and 10% fetal bovine serum. Cells were cultured in a humidified incubator at 37 °C with 5% CO_2_.

The Cell Counting Kit-8 (CCK-8) assay was employed to assess cell viability. Cells were seeded at an initial density of 2×10^4^ cells/well in a 96-well plate and incubated overnight at 37 °C. Cells were treated with varying concentrations of quercetin (1.25, 2.5, 5, 10, 20, 40, and 80 μmol/L) for 1, 3, and 5 days, respectively. Subsequently, cytotoxicity was assessed using CCK-8 following the manufacturer’s instructions. Based on the viability data, the median cytotoxic concentration (CC_50_) was calculated.

### Method details

#### Evaluating Anti-HBV activity of quercetin *in vitro*

HepG2.2.15 cells and HepG2.A64 cells were individually seeded in 48-well plates at a density of 2×10^4^ cells per well. Cells were treated with various drug concentrations: quercetin (0, 1.25, 2.5, and 5 μmol/mL) and TDF (0, 0.2, 2, 20, and 200 μmol/L), or selectively treated with specific quercetin concentrations for 5 days. On the 5th day, culture supernatants were collected to measure HBsAg and HBeAg levels using ELISA kits, and HBV DNA levels were determined by quantitative reverse - transcription PCR (qRT-PCR) assays. The half-maximal inhibitory concentration (IC_50_) and the selectivity index (SI, CC_50_/IC_50_) were determined.

#### Evaluating Anti-HBV activity of quercetin *in vivo*

C57BL/6 mice, adaptively fed, received injection of adeno-associated virus plasmid (pAAV) containing the 1.2-mer wild-type HBV genome (was kindly provided by Professor Dongping Xu, Institute of Infectious Diseases, Fifth Medical Center of Chinese PLA General Hospital, Beijing, China.) through tail vein hydrodynamic injection, applying pressure within 5-8 seconds and delivering 20 μg of plasmid. On the third day, blood samples were collected from the orbital vein to monitor levels of HBsAg, HBeAg, and HBV DNA.

The successfully modeled mice were divided into four groups, each consisting of six mice: the normal saline (NS) group, the low-dose quercetin (37.5 mg kg^−1^ d^−1^) group, the high-dose quercetin (75 mg kg^−1^ d^−1^) group, and the TDF (63 mg kg^−1^ d^−1^) group. Oral gavage was conducted daily for 4 weeks, and blood samples were collected weekly from the orbital vein to obtain serum. Levels of HBV DNA, HBsAg, and HBeAg were measured with ELISA kits following the manufacturer’s instructions. At the end of treatment, liver tissues were harvested for immunohistochemical (IHC) analysis of HBcAg and HBsAg expression. Quantitative analysis of IHC staining was performed using ImageJ software. HBsAg- and HBcAg-positive hepatocytes were analyzed under identical parameter and threshold settings for all samples, and the average density of antigen-positive hepatocytes was calculated for comparison among groups.

#### Evaluation of quercetin in the HBV models of RIG-I knockdow*n in vitro* and *vivo*

At the cellular level, three different siRNA sequences targeting RIG-I were designed. Each siRNA was transfected into cells at a final concentration of 20 nM, and cells were collected 48 hours post-transfection. RIG-I mRNA expression was subsequently assessed by qRT-PCR, and protein expression was analyzed via Western blot. The siRNA with the most effective knockdown was selected for subsequent functional experiments.

The LV-RIG-I-RNAi virus (target sequence: CCCTGAGCAAAGACCCCAAC), using GV911 as the vector, was constructed. The HBV mouse model was established by hydrodynamic tail vein injection of an HBV-expression plasmid. Based on this model, the virus was administered via tail vein injection to generate HBV models with RIG-I knockdown *in vivo*. Using this model, mice were orally administered quercetin consecutively for four weeks. After treatment, mice were sacrificed, and liver and serum samples were collected for HBV-related assessments as described above.

#### Reagents and antibodies

Quercetin (HY-18085),SYBR Green quantitative real-time polymerase chain reaction (qPCR) Master Mix (Cat No. HY-K0522), and RT Master Mix for qPCR (Cat No. HY-K0510) were supplied by MedChemExpress (Monmouth Junction, NJ, USA). TRIzol reagent (Cat No. 260805) was purchased from Invitrogen (Carlsbad, CA, USA). Anti-m IRF7 (72073)、anti-h IRF7 (4920)、anti-m pIRF7 (24129) and anti-h pIRF7 (5184) were obtained by Cell Signaling Technology (Boston, USA). Anti-MAVs (66911-1-ig)、anti-RIG-I (20566-1-AP) were provided by Proteintech. Furthermore, anti-GAPDH (GTX100118) was purchased from GeneTex (San Antonio, USA).

#### Network pharmacology and molecular docking analysis

Potential targets of quercetin were identified from the TCMSP database and normalized using UniProt, whereas HBV-associated targets were acquired from the GeneCards database.The overlapping targets were then imported into the STRING database to construct a PPI network, which was visualized in Cytoscape. Network topology analysis was performed using the NetworkAnalyzer plugin, and key nodes were identified based on degree centrality and betweenness centrality, reflecting their regulatory importance within the network. Functional enrichment analyses, including Gene Ontology (GO) and Kyoto Encyclopedia of Genes and Genomes (KEGG) pathway analyses, were were performed using Metascape.

To further validate interactions, molecular docking was conducted. Briefly, the 2D structure of quercetin was retrieved from the PubChem database and converted into a 3D structure using ChemOffice. The three-dimensional structures of RIG-I, MAVS, and IRF7 were retrieved from the Protein Data Bank (PDB). Water molecules and co-crystallized ligands were removed from the protein structures using PyMOL. PDBQT files were then prepared with AutoDockTools, and docking grids were defined with 40 points in each of the x, y, and z dimensions and a spacing of 1 Å to cover the potential binding pockets. Molecular docking calculations were subsequently performed using AutoDock Vina. The conformation with the lowest binding free energy was then visualized and analyzed in PyMOL.

#### Quantitative real-time reverse transcription PCR (qRT-PCR)

TRIzol was employed to extract total RNA from cells or tissues following the manufacturer’s protocol. Subsequently, the extracted RNA was reverse-transcribed to cDNA using RT Master. Real-time PCR was conducted using the SYBR Green qPCR Master Mix kit. The specific primer sequences designed and utilized for real-time PCR are provided in Table 1.

**Table 1:** Primers for quantitative real-time PCR analysis

Gene	Type	Sequence (5′–3′)
Human IFN-β	forward	ATGACCAACAAGTGTCTCCTCC
	reverse	GGAATCCAAGCAAGTTGTAGCTC
Human IRF7	forward	TTGGCTCCTGAGAGGGCA
	reverse	TTGGTTGGGACTGGATCTGC
Human RIG-I	forward	CTGGACCCTACCTACATCCTG
	reverse	GGCATCCAAAAAGCCACGG
Human MAVS	forward	CAGGCCGAGCCTATCATCTG
	reverse	GGGCTTTGAGCTAGTTGGCA
Human si RIG-I	forward	CCACUUAAACCCAGAGACAAUTT
	reverse	AUUGUCUCUGGGUUUAAGUGGTT
Human GAPDH	forward	AATGGGCAGCCGTTAGGAAA
	reverse	GCCCAATACGACCAAATCAGAG
Mouse IFN-β	forward	CAGCTCCAAGAAAGGACGAAC
	reverse	GGCAGTGTAACTCTTCTGCAT
Mouse IRF7	forward	GAGACTGGCTATTGGGGGAG
	reverse	GACCGAAATGCTTCCAGGG
Mouse RIG-I	forward	AAGAGCCAGAGTGTCAGAATCT
	reverse	AGCTCCAGTTGGTAATTTCTTGG
Mouse MAVS	forward	CTGCCTCACAGCTAGTGACC
	reverse	CCGGCGCTGGAGATTATTG
Mouse GAPDH	forward	AGGTCGGTGTGAACGGATTTG
	reverse	GGGGTCGTTGATGGCAACA

Relative gene expression levels were calculated using the 2^-ΔΔCT^ method, with GAPDH serving as the internal reference gene for normalization. Briefly, ΔCt was calculated as Ct_target − Ct_GAPDH, and ΔΔCt was calculated as ΔCt_treatment − ΔCt_control. GAPDH is a commonly used housekeeping gene in HBV-related cell models and liver tissues and has been extensively validated in previous studies to exhibit stable expression under comparable experimental conditions. All qPCR reactions were performed under identical amplification conditions to ensure comparable amplification efficiency across samples. Each experiment included at least three independent biological replicates (n = 3).

#### Western blot analysis

Liver tissue (50 mg) was homogenized to 1 mL of RIPA lysis buffer containing a protease inhibitor and homogenized at 100 Hz for 90 s. The tissue lysate was incubated on ice for 30 min, and the supernatant was then collected. The cell samples were lysed directly with RIPA lysis buffer. The protein concentration of all lysates was quantified using a BCA assay. Samples were boiled at 105°C for 15 minutes and separated by SDS-PAGE. Subsequently, the gels were transferred to a PVDF membrane, blocked with 5% skimmed milk for 1 h and then incubated with primary and secondary antibodies sequentially. Signals were analyzed using an enhanced chemiluminescence reagent and developed on film.

#### Surface plasmon resonance (SPR)

The binding interaction between quercetin and human RIG-I was analyzed by surface plasmon resonance (SPR) using a Biacore 8K system (Cytiva).Recombinant RIG-I was immobilized on a CM5 sensor chip using standard amine-coupling chemistry, with a reference flow cell left blank to correct for nonspecific binding, yielding an immobilization level of approximately 12,035 RU. Experiments were conducted in PBS-P+ buffer (pH 7.4) supplemented with 5% DMSO. Quercetin was injected at concentrations ranging from 0.3125 to 10 μM at a flow rate of 30 μL/min, and the sensor surface was regenerated with 10 mM glycine-HCl (pH 2.0) after each cycle. Sensorgrams were analyzed using Biacore Insight Evaluation software, and kinetic parameters (Ka, Kd) and equilibrium dissociation constant (KD) were calculated by global fitting to a 1:1 Langmuir binding model.

### Quantification and statistical analysis

All statistical analyses were performed using GraphPad Prism 9 (GraphPad Software, San Diego, CA, USA). Data are presented as the mean ± standard deviation (SD), where the mean represents the measure of central tendency and SD indicates the dispersion of the data. For comparisons between two groups, an unpaired two-tailed Student’s *t* test was used. For comparisons among three or more groups, one-way analysis of variance (ANOVA) followed by the appropriate post hoc multiple-comparisons test was performed, as indicated in the corresponding figure legends. The exact value of n for each experiment is provided in the figure legends. In *in vitro* experiments, n represents the number of independent biological replicates. In *in vivo* experiments, n represents the number of animals per group. Statistical details for each experiment, including the statistical test used, exact value of n, and significance definition, are provided in the figure legends. A value of p < 0.05 was considered statistically significant. Statistical significance is indicated as follows: ∗*p* < 0.05 and ∗∗*p* < 0.01; ns, not significant.
